# Detection of statistical asymmetries in non-stationary sign time series: Analysis of foreign exchange data

**DOI:** 10.1371/journal.pone.0177652

**Published:** 2017-05-18

**Authors:** Arthur Matsuo Yamashita Rios de Sousa, Hideki Takayasu, Misako Takayasu

**Affiliations:** 1 Department of Mathematical and Computing Science, School of Computing, Tokyo Institute of Technology, Midori-ku, Yokohama, Japan; 2 Sony Computer Science Laboratories, Shinagawa-ku, Tokyo, Japan; 3 Advanced Data Analysis and Modeling Unit, Institute of Innovative Research, Tokyo Institute of Technology, Midori-ku, Yokohama, Japan; East China University of Science and Technology, CHINA

## Abstract

We extend the concept of statistical symmetry as the invariance of a probability distribution under transformation to analyze binary sign time series data of price difference from the foreign exchange market. We model segments of the sign time series as Markov sequences and apply a local hypothesis test to evaluate the symmetries of independence and time reversion in different periods of the market. For the test, we derive the probability of a binary Markov process to generate a given set of number of symbol pairs. Using such analysis, we could not only segment the time series according the different behaviors but also characterize the segments in terms of statistical symmetries. As a particular result, we find that the foreign exchange market is essentially time reversible but this symmetry is broken when there is a strong external influence.

## Introduction

The development of new technologies allowed the extraction, storage and manipulation of large amount of data in diverse fields of knowledge, the so-called big data [1]. With this available volume of data, the new challenge is on how to process it and capture useful information about the system being studied. Econophysics is a relatively new research field that largely explores big data to access the characteristics and construct models in order to improve our understanding of economic systems. It applies methods traditionally developed in the scope of physics together with established economics ideas, strongly relying on statistical analysis of data [2–4]: Analysis of scaling properties [5–7], analogies with physical systems as the Brownian particle [8–11] and the Ising model [12, 13], studies related to equilibrium as in thermodynamics [14–16]. Aside the name, econophysics has become a broader discipline, also using concepts from other areas as network theory [17–19] and information theory [20–22].

In this paper, we analyze time series data of the foreign exchange market, known to be non-stationary. The foreign exchange market is the financial market for trading of currencies that defines their relative values and affects other markets, e.g., the stock markets [23]. The analysis of such data and its interpretation is a challenge since it is the output of a complex system composed by several interacting agents that also interact with the external environment. Here we characterize different intervals of the time series using the concept of statistical symmetry. The statistical symmetry refers to the invariance of a probability distribution under some transformation; by taking different transformations, we can obtain information on different aspects of the market price movements according to the kind of symmetry being analyzed.

For the analysis of the market data, we take the signs of price changes and perform a hypothesis test to evaluate if the analyzed sequence of signs came from a symmetric Markov process or not for two types of symmetries: Independence and time reversion. We apply a sliding window local analysis in the binary sign time series to obtain the evolution of the behavior of the market regarding each symmetry; in particular, we are interested in periods when an external influence affects the market, e.g., a government intervention. A local analysis is particularly important for non-stationary time series such as financial series, for which a single global characterization may hide useful information about the system.

The paper is structured as follows. First, we present the concept of statistical symmetry and describe the hypothesis test to analyze real data using the statistical symmetries of independence and time reversion and the Markov process as model. Then, we detail the data of the U.S. Dollar and Japanese Yen (USD/JPY) market and present our results, showing the segmentation of the time series in symmetric and asymmetric intervals for each symmetry and discussing the period when the Japanese government intervened in the USD/JPY market as well as other periods characterized by important asymmetries. Finally, we conclude summarizing the framework and obtained results and mentioning the perspectives.

## Statistical symmetry

Symmetry can be defined as invariance under some transformation. As a simple and familiar example, we have a square with 90° rotation symmetry because it remains exactly the same if we rotate it by multiples of 90°. However, the invariance can be not exact/deterministic; in this case we say statistical symmetry. A known example of process with statistical symmetry is the random walk, which has statistical scale symmetry: When we apply an appropriate scale transformation to its path, it does not remain exactly the same but its statistical properties remain invariant (it is a statistical fractal) [24]. The term ‘statistical symmetry’ is not new; besides fractal geometry, it has been used in statistical physics and hydrodynamics to describe symmetries of average quantities [25, 26]. Here we extend this concept to deal with transformations on probability distributions, defining statistical symmetry as the invariance of a probability distribution under some transformation.

Consider the transformation *T* on the probability distribution *P*: $\tilde{P}=T\left[P\right]$. We can quantify the degree of symmetry of the probability distribution *P* in respect to the transformation *T* by using the Kullback-Leibler divergence, also called relative entropy [27]. The Kullback-Leibler divergence between *P* and $\tilde{P}$, considering the possible events ***X*** in the discrete sample space Ω, is defined as:

$$\begin{array}{c} D(P||\tilde{P})=\sum_{X}P\left(X\right)\log\frac{P(X)}{\tilde{P}\left(X\right)};\;X\in \Omega . \end{array}$$ (1)

The symmetric distribution *P*^⋆^ corresponding to the transformation *T* is such that *P*^⋆^ = *T*[*P*^⋆^]and for it *D*(*P*^⋆^||*T*[*P*^⋆^]) = 0. Because of the property of the Kullback-Leibler divergence, *D* is equal to 0 only for the symmetric distribution of the given transformation *T*.

By using the Kullback-Leibler divergence, we recover quantities from the information theory and statistical physics and can properly interpret some statistical symmetries, as we illustrate next for two particular types of transformation:

(1) Independence transformation: For an appropriate sample space, it transforms a probability distribution in the product of the marginal probability distributions. In the space $\Omega =R^{2}$:

$$\begin{array}{c} \tilde{P}\left(x,y\right)=P\left(x\right)P\left(y\right). \end{array}$$ (2)

The Kullback-Leibler divergence is given by:

$$\begin{array}{c} D\left(P\left(x,y\right)||P\left(x\right)P\left(y\right)\right)=\sum_{x,y}P\left(x,y\right)\log\frac{P(x,y)}{P(x)P(y)}, \end{array}$$ (3)

which is the definition of mutual information from information theory, evidencing that the independence symmetry indeed informs about the independence between random variables.

(2) Reversion: For an appropriate sample space, it assigns the probability of a sequence of events to the probability of the corresponding reversed sequence.

$$\begin{array}{c} \tilde{P}\left(X\right)=P\left(X^{†}\right);\;X^{†}=AX, \end{array}$$ (4)

where transformation *A* is a reversion. For example, in the space $\Omega =R^{n}$, if *A* is an even reversion: (*x*_1_, …, *x*_*n*_) → (*x*_*n*_, …, *x*_1_); if *A* is an odd reversion (*x*_1_, …, *x*_*n*_) → (−*x*_*n*_, …, −*x*_1_).

If the sequence of random variables (*x*_1_, …, *x*_*n*_) refers to a time evolution, the reversion symmetry can be regarded as time reversibility since it tells if the probability of the forward sequence of variables is equal to the probability of the corresponding backward one—even (odd) reversion for even (odd) variables as function of time.

We express the Kullback-Leibler divergence in this case by:

$$\begin{array}{c} D(P\left(X\right)||P\left(X^{†}\right))=\sum_{x_{i}}P\left(x_{1},...,x_{n}\right)\log\frac{P(x_{1},...,x_{n})}{P(\pm x_{n},...,\pm x_{1})}, \end{array}$$ (5)

which is a measure of entropy production studied in the area of non-equilibrium physics [28–30], revealing the connection between statistical time reversion symmetry and entropy production.

Statistical symmetries can be used to characterize a stochastic process model, with its evolution governed by an underlying probability distribution. Let us investigate statistical symmetries in a binary sign time homogeneous Markov process, a simple model used in the next section to describe the analysis of sign time series. In this process, the possible events are in *B* = {+, −} and the next symbol only depends on the previous one:

$$\begin{array}{c} P\left(x_{n+1}|x_{1},x_{2},...,x_{n}\right)=P\left(x_{n+1}|x_{n}\right);\;x_{i}\in B, \end{array}$$ (6)

where *P*(*e*_2_|*e*_1_) is the conditional probability of event *e*_2_ given *e*_1_.

The transition matrix ***T*** of such Markov process is:

$$\begin{array}{c} T=[\begin{array}{cc} 1-\mu & \nu \\ \mu & 1-\nu \end{array}], \end{array}$$ (7)

where *μ* and *ν* represent the transition probabilities (constant for all *n*, since we are considering a time homogeneous process):

$$\begin{array}{c} \mu =P\left(x_{n+1}=-|x_{n}=+\right)=1-P\left(x_{n+1}=+|x_{n}=+\right), \end{array}$$ (8)

$$\begin{array}{c} \nu =P\left(x_{n+1}=+|x_{n}=-\right)=1-P\left(x_{n+1}=-|x_{n}=-\right). \end{array}$$ (9)

One step in the Markov process is given by:

$$\begin{array}{c} P_{n+1}=TP_{n}, \end{array}$$ (10)

with

$$\begin{array}{c} P_{n}=[\begin{array}{c} P(x_{n}=+)\\ P(x_{n}=-) \end{array}]. \end{array}$$ (11)

The process is in its stationary state when ***P***_*n*+1_ = ***P***_*n*_ = ***P***, i.e., ***P*** = ***TP***, for which we have:

$$\begin{array}{c} P\left(x=+\right)=1-P\left(x=-\right)=\frac{\nu }{\mu +\nu }. \end{array}$$ (12)

**Fig 1:**
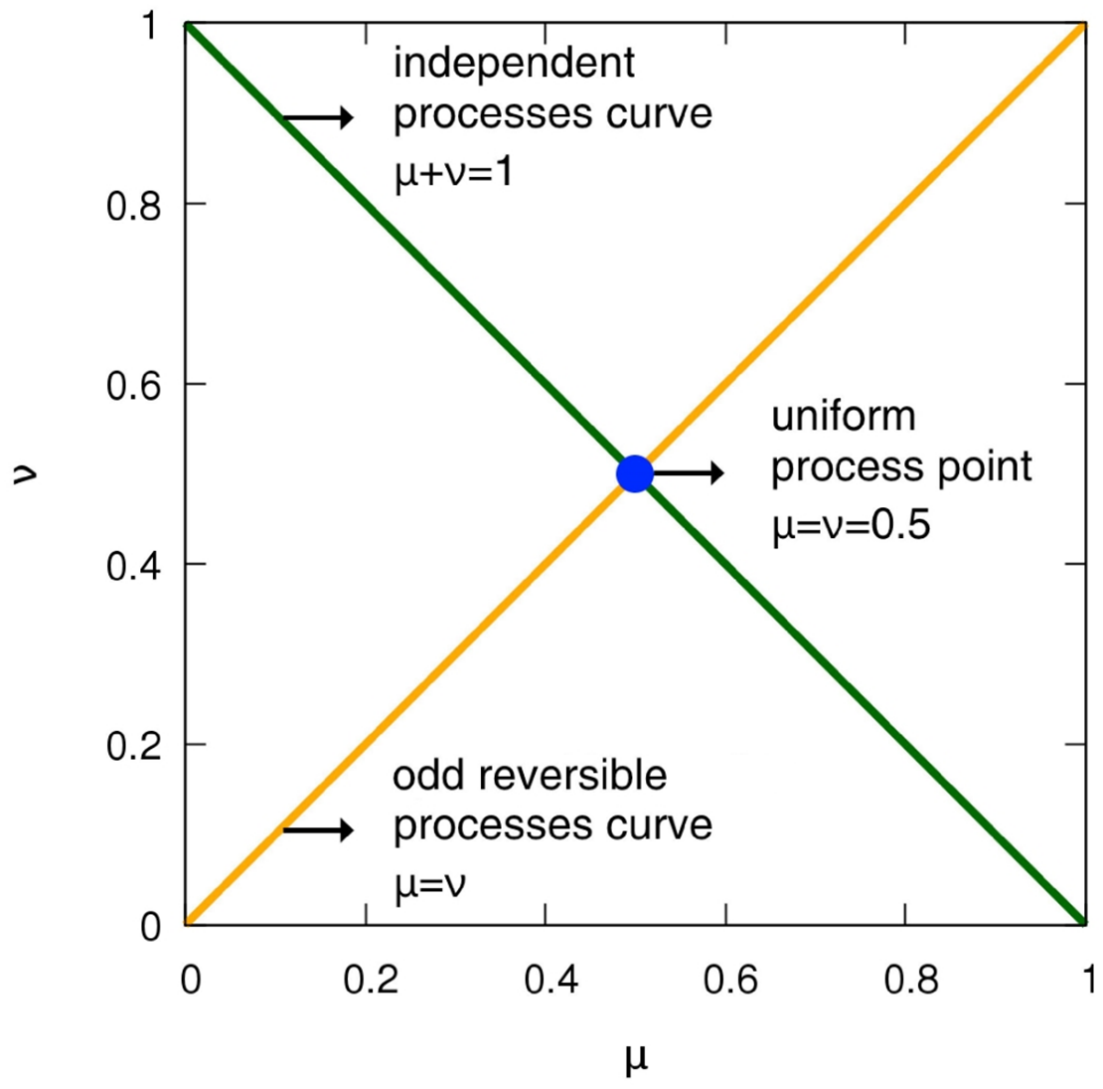
Statistically symmetric process curves in the parameter space of binary Markov process. Independence transformation symmetry (green) and odd reversion symmetry (orange); the uniform process is the only intersection between independent processes and reversible processes.

Considering the stationary state, we can determine the values of *μ* and *ν* corresponding to the symmetric process of both transformations we defined previously (see Fig 1):

(1) For the independence transformation, the parameters of the symmetric probability distribution satisfy the condition for the joint probability *P*(*x*_*n*+1_ = +, *x*_*n*_ = +) = *P*(*x* = +)*P*(*x* = +), for all *n*, resulting in:

$$\begin{array}{c} \mu +\nu =1. \end{array}$$ (13)

(2) For the odd reversion, it is enough for the symmetric process to have *P*(*x*_*n*+1_ = +, *x*_*n*_ = +) = *P*(*x*_*n*+1_ = −, *x*_*n*_ = −), for all *n*. The values of *μ* and *ν* satisfy:

$$\begin{array}{c} \mu =\nu , \end{array}$$ (14)

which also implies *P*(*x* = +) = *P*(*x* = −). Note that a stationary sign binary Markov process is always even reversion symmetric, i.e., *P*(*x*_*n*+1_ = +, *x*_*n*_ = −) = *P*(*x*_*n*+1_−, *x*_*n*_ = +).

## Analysis of sign time series and hypothesis test

In general, we do not know the process that generated a given time series and then we do not have access to the true probability distribution to study its statistical symmetries. In such case, we need a procedure to evaluate if time series is symmetric or not for different transformations; we describe next a hypothesis test in sign time series for this purpose.

We assume that a segment of finite length *w* of the sign time series can be modeled as a stationary sign binary Markov process (described in the previous section) and set the symmetric Markov process corresponding to the transformation we are examining as the null hypothesis *H*_0_ and a corresponding asymmetric process as the alternative hypothesis *H*_1_. Characterizing a sign binary Markov process by the transition probabilities *μ* and *ν*, as defined above, we decide if we accept or reject *H*_0_ based on the probability distribution of such Markov process {*μ*, *ν*} in its stationary state to generate a sequence of given length *w* with a set of specific number of each pair of symbols, *N*_++_, *N*_+−_, *N*_−+_, *N*_−−_, which is taken as the test statistic. We use pairs of symbols because this information is enough to compute the probability of a specific sequence of a Markov process. Moreover, we would only need the probabilities of pairs of symbols to determine the parameters *μ* and *ν* of the Markov process and thus its statistical symmetries.

The probability P of a stationary sign binary Markov process {*μ*, *ν*} to generate a sequence with numbers of pairs *N*_++_, *N*_+−_, *N*_−+_, *N*_−−_ and a given boundary is:

$$\begin{array}{c} P=P(N_{++},N_{+-},N_{-+},N_{--},s_{1},s_{w}|\left\{\mu ,\nu \right\};w)=\gamma \Phi , \end{array}$$ (15)

where *s*_1_ (*s*_*w*_) represents the first (last) symbol of the sequence. Here, *γ* is the combinatorial factor for pairs of symbols in a binary sequence [31–33] (S1 Appendix):

γ={1;ifN+=0orN+=w(N+-1N++)(N--1N--);if0<N+<w, (16)

with *N*_+_ (*N*_−_) indicating the number of symbols (+) ((−)), and Φ is the probability of the considered Markov process {*μ*, *ν*} to generate a specific sequence:

$$\begin{array}{ccc} \Phi & = & {\left(\frac{\nu }{\mu +\nu }\right)}^{\varepsilon (+,+)+\varepsilon (-,+)}{\left(\frac{\mu }{\mu +\nu }\right)}^{\varepsilon (+,-)+\varepsilon (-,-)}\\ & & \times {\left(1-\mu \right)}^{N_{++}}\mu^{N_{+-}}\nu^{N_{-+}}{\left(1-\nu \right)}^{N_{--}}, \end{array}$$ (17)

where *ε*(*i*, *j*) is the boundary indicator of the sequence, with *i*, *j* = (+) or (−):

$$\begin{array}{c} \varepsilon \left(i,j\right)=\{\begin{array}{c} 1;\;\text{if}\;s_{w}=i\;\text{and}\;s_{1}=j\\ 0;\;\text{otherwise}\; \end{array}. \end{array}$$ (18)

The numbers of each pair in a binary sequence are not independent [34]; in fact, we can obtain the other numbers of pairs by knowing *N*_+_, *N*_++_ and the boundary of the sequence (S1 Appendix). Applying the normal approximation in Eq (12), we derive a computationally feasible expression for the probability distribution of numbers of pairs (S2 Appendix), which for appropriate values of parameters *μ* and *ν* constitutes the distribution of the test statistics under *H*_0_:

$$\begin{array}{ccc} P & = & P\left(N_{+},N_{++},s_{1},s_{w}|\left\{\mu ,\nu \right\};w\right)\approx \frac{\nu^{1+\varepsilon (+,+)-\varepsilon (-,-)}\mu^{1-\varepsilon (+,+)+\varepsilon (-,-)}}{\mu +\nu }\\ & & \times \frac{1}{\sqrt{2\pi \left(N_{+}-1\right)\mu \left(1-\mu \right)}}exp\left(-\frac{{\left[N_{++}-\left(N_{+}-1\right)\left(1-\mu \right)\right]}^{2}}{2\left(N_{+}-1\right)\mu \left(1-\mu \right)}\right)\\ & & \times \frac{1}{\sqrt{2\pi \left(N_{-}-1\right)\nu \left(1-\nu \right)}}exp\left(-\frac{{[\left(N_{--}-\left(N_{-}-1\right)\left(1-\nu \right)\right]}^{2}}{2\left(N_{-}-1\right)\nu \left(1-\nu \right)}\right), \end{array}$$ (19)

with *N*_−_ = *w* − *N*_+_ and *N*_−−_ = *w* − 2*N*_+_ + *N*_++_ + *ε*(+, +) − *ε*(−, −).

The hypothesis test, therefore, consists in computing the probability P for the sign sequence we are analyzing. We choose the parameters of the Markov process *μ* and *ν* that refer to the symmetric process of the statistical symmetry we would like to examine: a independence symmetric process has parameters such that *μ* + *ν* = 1 and a time reversible process presents parameters obeying *μ* = *ν*. Because there are infinite parameters *μ* and *ν* satisfying each of those symmetries, we select the ones that result in the maximum probability P, giving an upper bound for the probability of the symmetric process to generate the analyzed sequence. To decide if we accept or reject *H*_0_, we compare the calculated probability to a threshold probability *z* and use the rule: If P≥z, we accept the *H*_0_ and the process that generated the sign sequence is evaluated as symmetric; if P<z, we reject it. We choose the value *z* = 10^−7^ for the probability threshold in order to detect asymmetries. From Markov process simulations (S3 Appendix), this value of *z* corresponds to a probability below 10^−4^ of evaluating a symmetric interval as an asymmetric one. On the other side, the probability of evaluating an asymmetric interval as a symmetric one depends on the strength of asymmetry (i.e., how much the parameters characterizing a process deviate from the conditions *μ* + *ν* = 1 for independence symmetry or *μ* = *ν* for time reversion symmetry): The probability is large for weak asymmetry, not being detected by the test, but the probability is around 0.8 for mild asymmetry and approaches 0 for strong asymmetry. Thus, such uncommon low threshold extremely reduces the detection of false asymmetries, but still allow us to detect mild and strong asymmetries.

The analysis of independence symmetry for sign sequences is related to the Wald–Wolfowitz runs test, used to decide if the elements of a binary sequences are mutually independent [35, 36]. We comment this relationship in S4 Appendix.

## Foreign exchange market data

We analyze the foreign exchange data of the Electronic Broking Service (EBS) by ICAP. This market is continuously open during the weekdays and it has the largest transaction volume among the financial markets. Traders can make orders for buying and selling for a given currencies pair which are organized in the order book according to their corresponding prices. A deal occurs when the highest buying order and the lowest selling order meet each other and the mid-quote is defined at each time as the average of those two orders. The data contains the orders for pairs of currencies in original sampling time of 0.1*s* forming time series. We take the sign of the change in the mid-quote, resulting in a series containing the symbols: (+) (price increasing), (−) (price decreasing) and 0 (no change in price). We directly remove the zeros, resulting in a binary series of (+) and (−). Removing the zeros means that we are no longer working with the clock time but only when there is an increase or a decrease in mid-quote, which is generally called tick time. Also, analyzing the sign time series represents a reduction of information since we remove the data about the magnitude of the changes; however, we still keep the important information about the price change direction [37, 38].

**Fig 2:**
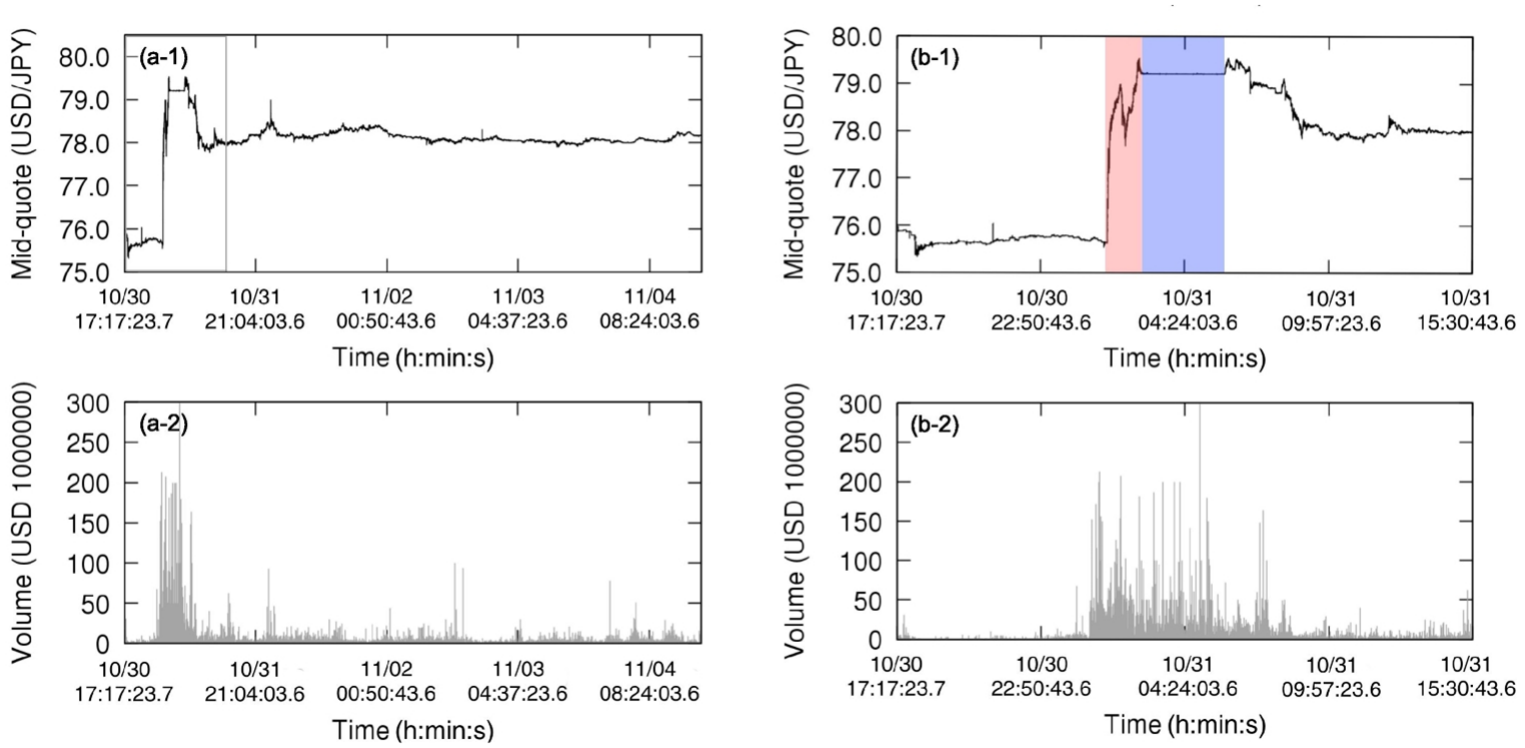
Time series of mid-quote of the pair U.S. dollar (USD) and Japanese yen (JPY) in the foreign exchange market. (a-1) Time series of mid-quote of USD/JPY from 2011, October, 30th to 2011, November, 5th in original sampling time of 0.1*s* (clock time). (a-2) Corresponding time series of volume of orders. (b-1) Zoomed graph—gray box in (a-1)—shows when the Japanese government intervened in the market on October, 31st; the action of the Japanese government can be divided in two phases, first causing a quick appreciation of the U.S. dollar (from GMT 1h25 to 2h40—red box) followed by an almost constant price period (from GMT 2h40 to 5h55—blue box). (b-2) Corresponding time series of volume of orders, with a high number of orders during the period under the effect of the intervention.

We focus on sign time series of the pair U.S. dollar and Japanese yen (USD/JPY) in October and November of 2011, from October, 2nd to December, 3rd (9 weeks). During this period there was the Japanese government intervention in the market on October, 31st, selling Japanese Yen to weaken the currency expecting increase of exports [39]. This intervention was implemented in two phases: The first one to shift the mid-quote to a higher value and the second one to stabilize the price (see Fig 2). We especially pay attention to this event in order to observe the effect of exogenous agents in the foreign exchange market that significantly change its behavior.

## Statistical symmetries for sign time series of USD/JPY

We apply the presented hypothesis test to the sign time series of the pair of currencies USD/JPY. We perform a local analysis in the sign time series through a sliding window procedure: In each window of length *w* we assume the time series can be described by a stationary sign binary Markov process and compute the probability P of a symmetric process; we can then classify each window as corresponding to a symmetric process or not. Modeling the time series of the sign of price changes as Markov process is a consistent assumption from sampling time 0.4*s*: It has been shown that the autocorrelation function for the price difference in this market goes to zero after few ticks [40]; also in S5 Appendix we observe that the auto mutual information [41] from the mentioned sampling time has similar behavior for the present data set. Results shown here refer to sampling time 0.4*s*.

For this particular sign time series, the independence symmetry tell us if a price movement (up or down) in the present depends or not on the past price movement, also evidencing dependence patterns (e.g., zigzag structures), while the time reversion symmetry inform us if the price movements tend to cancel each other or if there are trends in the price movement, possibly indicating a trend-following behavior of dealers.

**Fig 3:**
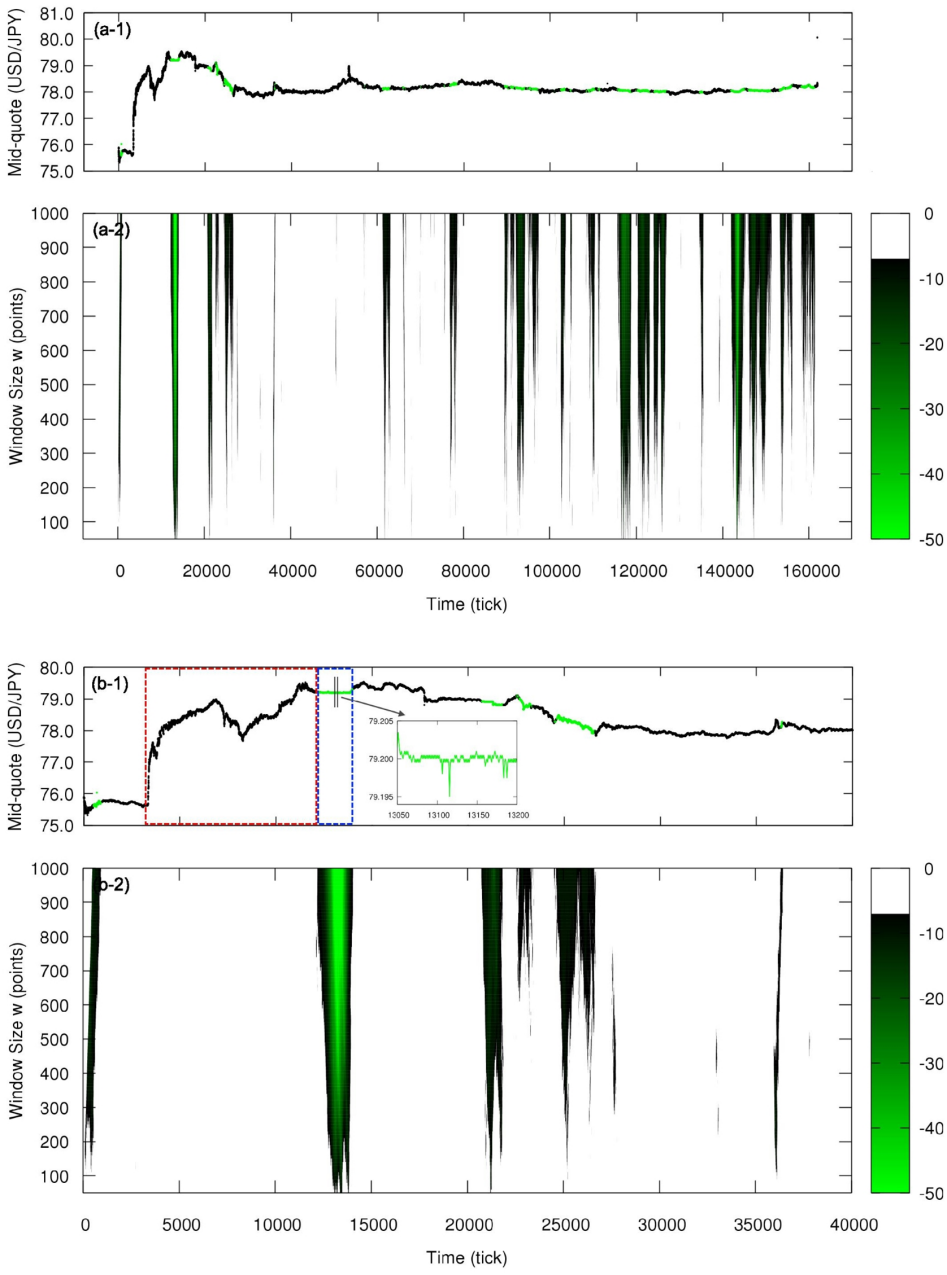
Local analysis of the independence statistical symmetry for the currency pair USD/JPY in the week from 2011, October, 30th to 2011, November, 5th. (a-1) Mid-quote time series in tick time with intervals evaluated as independence asymmetric shown in green (for window size *w* = 1000). (a-2) Color plot showing window size effect on the hypothesis test; color code refers to the decimal logarithm of the probability P and points are colored only if P is below the threshold 10^−7^, i.e., if they are classified as independence asymmetric. (b-1) and (b-2) details the Japanese government intervention; red box indicates the quick increase in the mid-quote and the blue box the period of almost constant mid-quote. The period corresponding to the increase in the mid-quote is classified as independence symmetric and the almost constant period, independence asymmetric.

In Fig 3 we show the results for the independence symmetry. Fig 3-a is the results for all the week from 2011, October, 30th to 2011, November, 5th, while Fig 3-b details the period of the intervention on the beginning of October, 30th. In Fig 3-a-1 and 3-b-1, we straightforwardly project the result of the hypothesis test on the mid-quote time series (in tick time, not clock time) by coloring each point according to the test in the corresponding window of size *w* = 1000 having the point as mid-point of the window: Black represents the acceptance of the symmetric null hypothesis and green indicates its rejection, considering the probability threshold 10^−7^. In Fig 3-a-2 and 3-b-2, we show the results of the hypothesis test for different window size *w* and also add the information about the value of the probability P: From the threshold 10^−7^, lighter the green, smaller is P (in decimal logarithm scale). Since the hypothesis acceptance-rejection results may be rough and criticism to the use of hypothesis test exist [42, 43], the visualization of the magnitude of P contributes to increase the belief in the rejection of symmetric process description if the probability is too small.

From Fig 3-b-1 and 3-b-2, we observe that the increase in the mid-quote caused by the first phase of the intervention (from tick ∼5000 until tick ∼25000) is in general classified as independent and the following almost constant price period caused by the second phase of the intervention is evaluated as not independent, with $P\approx 10^{-50}$. A possible explanation for the independence symmetry for the mid-quote increase is that it was caused mainly by the external influence represented by the intervention and not by influence of the past internal market movement, meaning that the traders during that period were not using the past changes in the market to decide their moves. For the almost constant price period, any change in the mid-quote was followed by the opposite change producing zigzag structures (see zoom in Fig 3-b-1), which are highly dependent, i.e., the next symbol strongly depends on the previous one. Besides those special periods, one possible factor that could change the dynamics of the process regarding the independence symmetry (from independent to dependent behavior, for example) is the scheduled data release by the governments and the central banks, eg., exports and imports balance and unemployment rate. Such data has a predetermined time to be announced and thus plays with the expectation and reaction of the traders. Other analyses are needed to verify this hypothesis and detect other factors.

**Fig 4:**
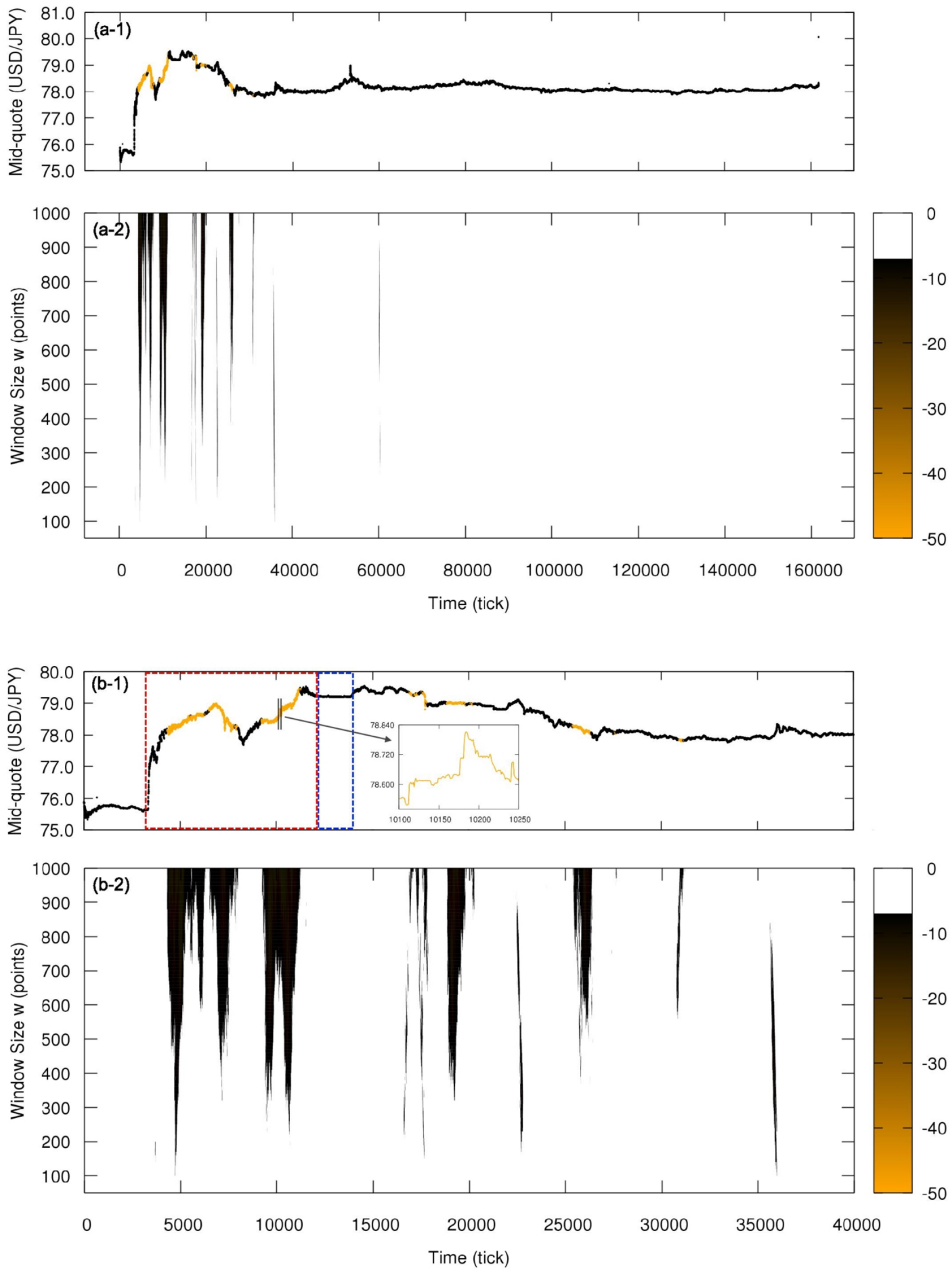
Local analysis of the time reversion statistical symmetry for the currency pair USD/JPY in the week from 2011, October, 30th to 2011, November, 5th. (a-1) Mid-quote time series in tick time with intervals evaluated as time reversion asymmetric shown in orange (for window size *w* = 1000). (a-2) Color plot showing window size effect on the hypothesis test; color code refers to the decimal logarithm of the probability P and points are colored only if P is below the threshold 10^−7^, i.e., if they are classified as time reversion asymmetric. (b-1) and (b-2) details the Japanese government intervention; red box indicates the quick increase in the mid-quote and the blue box the period of almost constant mid-quote. Time reversion asymmetric intervals only occur during the intervention and a short period right after it.

Fig 4 shows analogous results for the time reversion symmetry (here the color orange indicating rejection of null hypothesis). For this symmetry, we only observe deviations from the null hypothesis in some segments before tick ∼40000, corresponding to the intervention and a transition period; the almost constant mid-quote period—second phase of intervention—is time reversible because of the zigzag dynamics. From tick ∼40000, the market remains essentially time reversible until the end of the week, reflecting the absence of short-term tendencies in the mid-quote; in contrast, the period under the effect of the first phase of the intervention has several segments considered time irreversible presenting a strong tendency to change the mid-quote in a short period of time (see zoom in Fig 4-b-1). Note that the intervention was not implemented continuously but in steps, what can be verified in Fig 2-b-2 with the volume of orders presenting peaks and explains why not all windows during the intervention are time irreversible.

**Fig 5:**
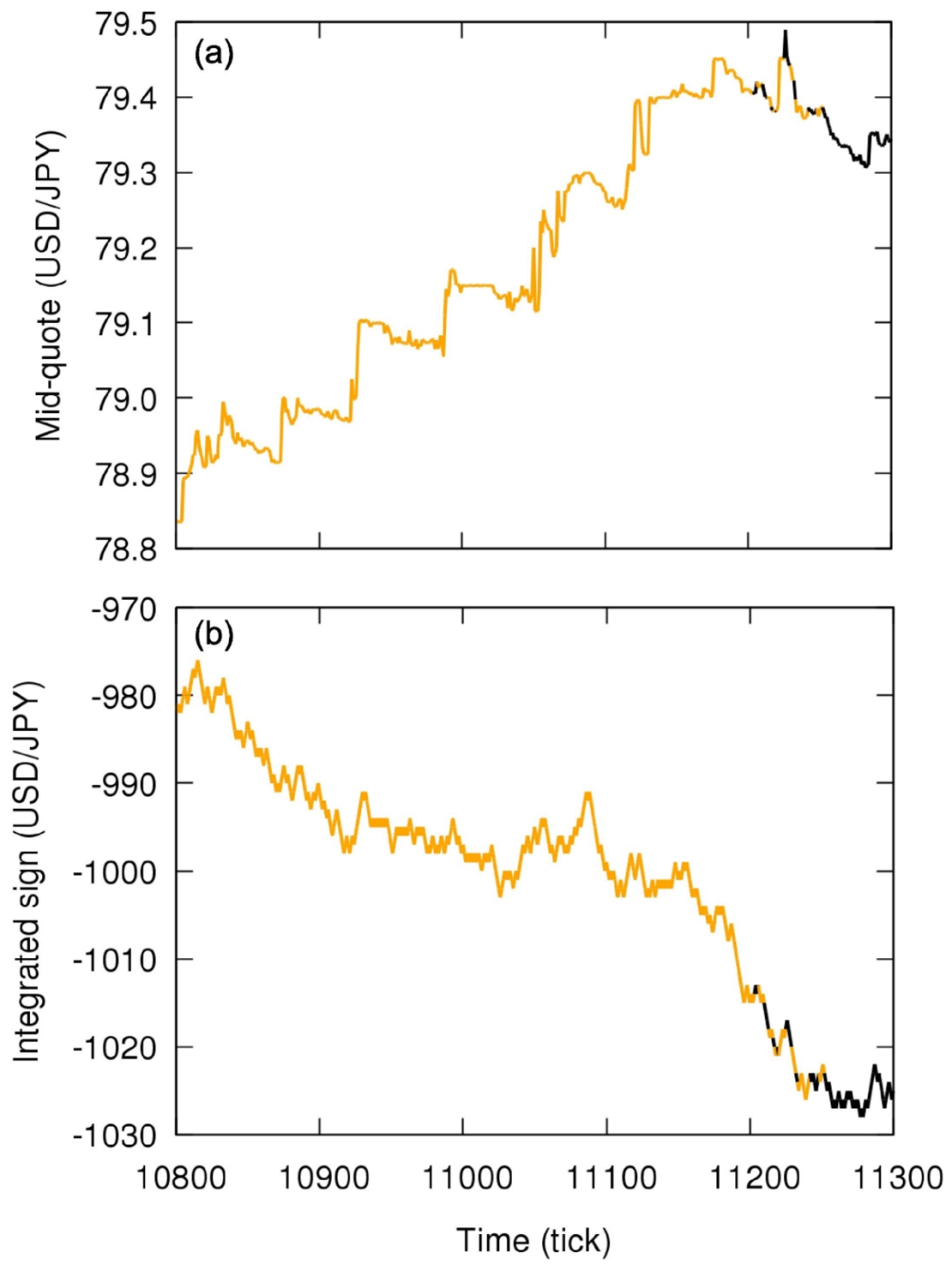
Details of local analysis of the time reversion statistical symmetry for price time series and corresponding price difference integrated sign time series for week from 2011, October, 30th to 2011, November, 5th. (a) Zoomed USD/JPY mid-quote time series in tick time with intervals evaluated as time reversion asymmetric shown in orange (for window size *w* = 1000). (b) Corresponding time series for the integrated sign of the price difference in the USD/JPY market. During the intervention period, local tendencies due to persistent movement of the price in one direction—and not due to changes of large magnitude—are opposite to the mid-quote general tendency.

Since we are analyzing only the sign of the price change and not its magnitude, the tendencies detect by the time reversion asymmetry are not due to changes of large magnitude, but they are consequence of several changes in one direction regardless the magnitude, reflecting the action of various traders rather than few ones. In fact, the quick increase in the mid-quote during the first phase of the intervention corresponds to few big changes by a limited number of traders (such as the Bank of Japan), not detectable by the applied time reversion asymmetry analysis; the local tendencies actually detected seem to be counteractions to the changes of large magnitude and are in the opposite direction of the mid-quote general tendency (see Fig 5).

**Fig 6:**
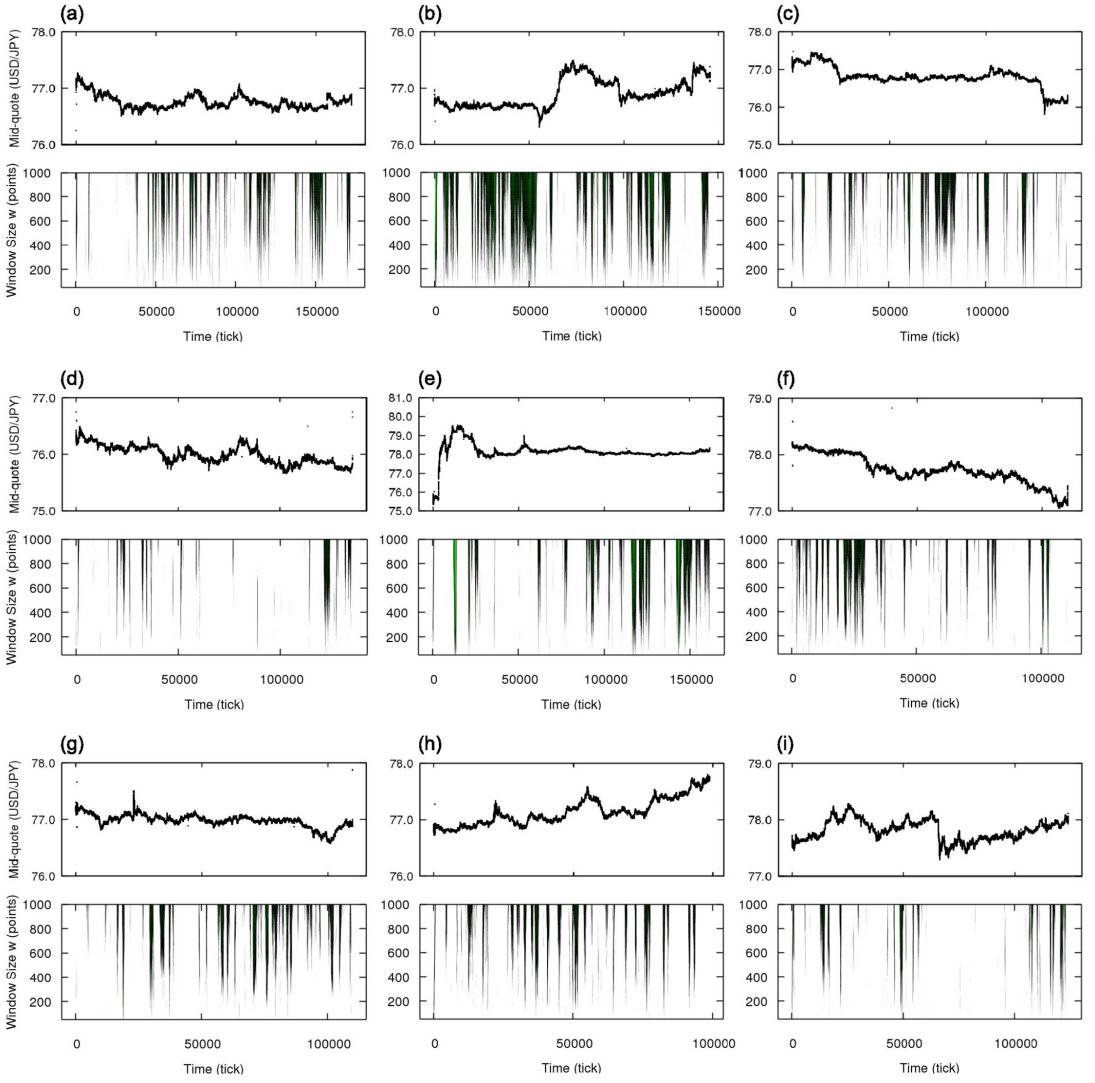
Mid-quote time series in tick time and local analysis of the independence statistical symmetry for the currency pair USD/JPY in nine weeks of October and November of 2011. (a) from October, 2nd to October, 8th, (b) from October, 9th to October, 15th, (c) from October, 16th to October, 22nd, (d) from October, 23rd to October, 29th, (e) from October, 30th to November, 5th, (f) from November, 6th to November, 12th, (g) from November, 13th to November, 19th, (h) from November, 20th to November, 26th and (i) from November, 27th to December, 3rd.

**Fig 7:**
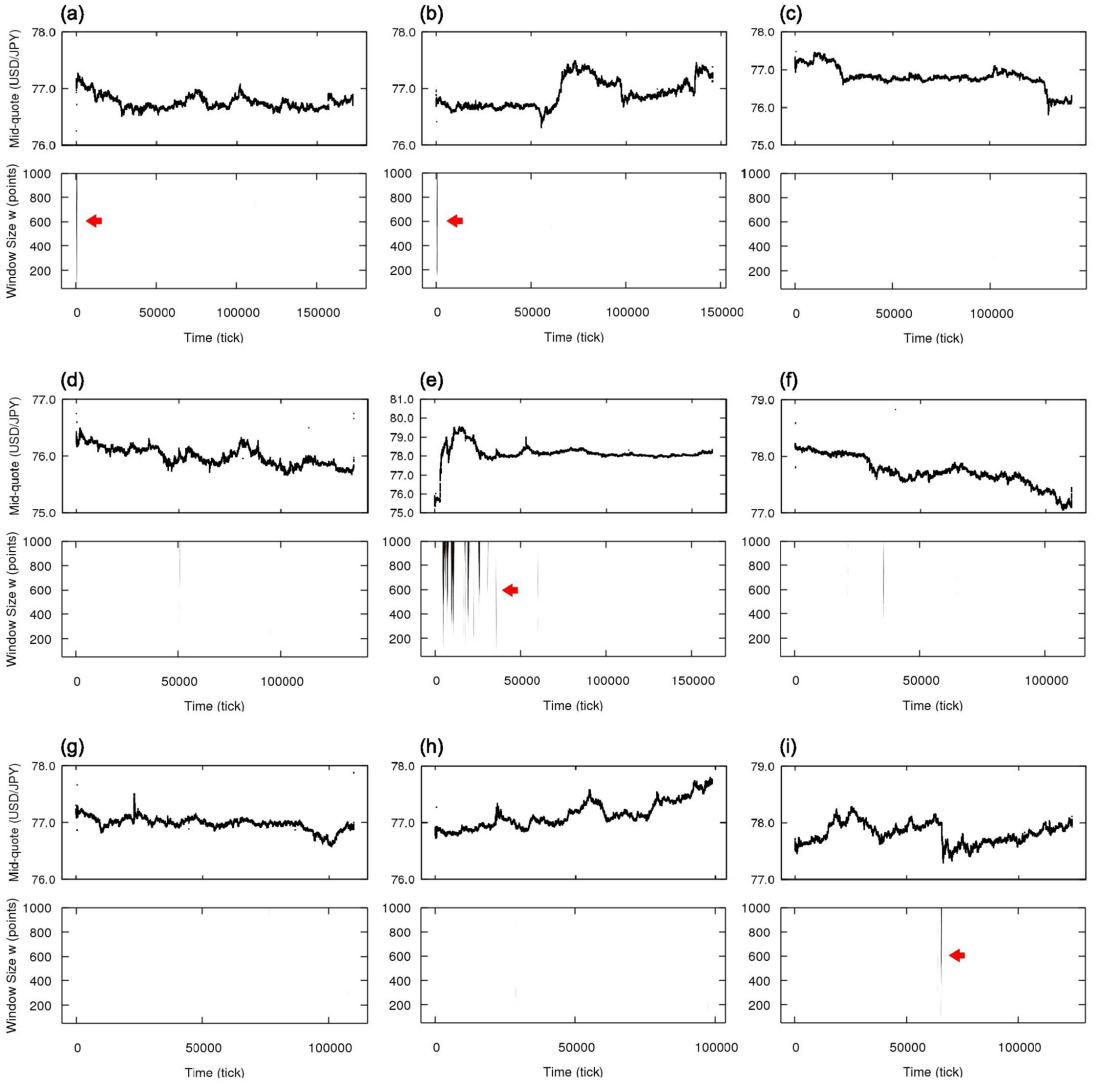
Mid-quote time series in tick time and local analysis of the time reversion statistical symmetry for the currency pair USD/JPY in nine weeks of October and November of 2011. (a) from October, 2nd to October, 8th, (b) from October, 9th to October, 15th, (c) from October, 16th to October, 22nd, (d) from October, 23rd to October, 29th, (e) from October, 30th to November, 5th, (f) from November, 6th to November, 12th, (g) from November, 13th to November, 19th, (h) from November, 20th to November, 26th and (i) from November, 27th to December, 3rd. All weeks are characterized mainly as time reversion symmetric, except during special intervals where significant deviations from the symmetric hypothesis for several window sizes appear (indicated by red arrows), in particular four occasions: In weeks (a) and (b), the symmetry breaking occurs in a small interval in the beginning of week, when the foreign exchange market opens; in week (e), there are several asymmetric intervals during the Japanese government intervention; and in week (i) there is one short asymmetric period corresponding to the announcement of global central bank intervention.

**Fig 8:**
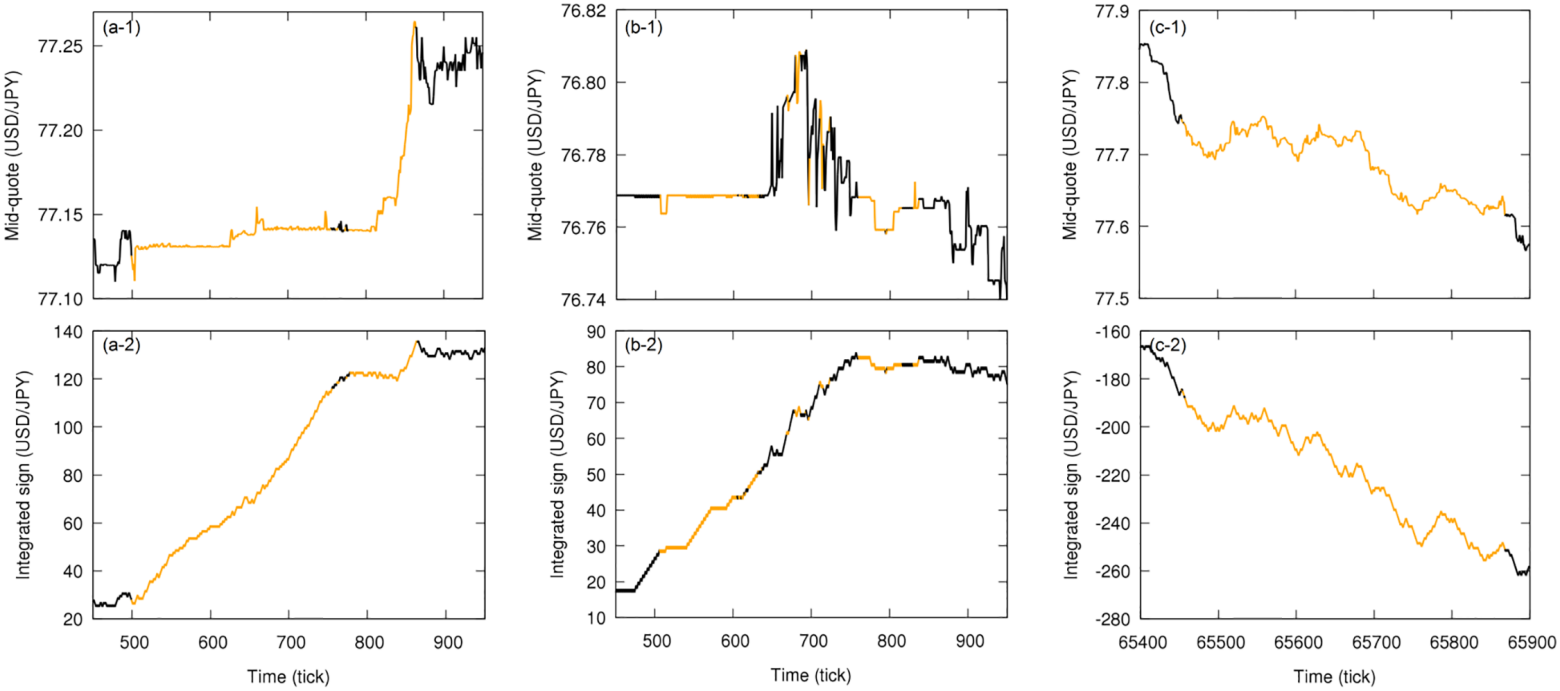
Details of local analysis of the time reversion statistical symmetry for price time series and corresponding price difference integrated sign time series for weeks from 2011, October, 2nd to October, 8th, from October, 9th to October, 15th and from November, 27th to December, 3rd. Zoomed USD/JPY mid-quote time series in tick time with intervals evaluated as time reversion asymmetric shown in orange (for window size w = 1000) for week (a-1) from October, 2nd to October, 8th, (b-1) from October, 9th to October, 15th and (c-1) from November, 27th to December, 3rd. Corresponding time series for the integrated sign of the price difference in the USD/JPY market for week (a-2) from October, 2nd to October, 8th, (b-2) from October, 9th to October, 15th and (c-2) from November, 27th to December, 3rd. The first two asymmetric periods occur in the beginning of the respective weeks and present similar trend with zigzag structures, while the last asymmetric interval shows local tendencies with prices moving in a preferred direction that agrees with the mid-quote general tendency.

In order to further investigate the independence and time reversion symmetries in the market data, we analyze eight more weeks of October and November of 2011, from October, 2nd to December, 3rd. In Fig 6, we show the results for the independence symmetry with the identification of independent and dependent intervals. A different result is found for the time reversion symmetry: From the results in Fig 7, the general behavior of the market appears to be characterized as time reversible. For the considered data, however, we find some localized intervals in which this symmetry is broken, particularly during four events. The first two occur in the beginning of weeks from October, 2nd to October, 8th and from October, 9th to October, 15th (Fig 7-a and 7-b). Those two events are not only similar in respect to the time of the week they happen but they also present similar sharp trend with zigzag structures (see Fig 8-a and 8-b), characterizing both strong independence asymmetry and time irreversibility; the exact cause for those asymmetries are not clear but they are related to the particular behavior of active dealers when the market is opening, when there are fewer traders and less liquidity, being rather a structural effect than an effect of the market dynamics. The next one corresponds to the already discussed Japanese government intervention on October, 30th (Fig 7-e). The last one happens in November, 30th (Fig 7-i) and represents the reaction of the market to the announcement of the coordinated central bank intervention by the Bank of Canada, the Bank of England, the Bank of Japan, the European Central Bank, the Federal Reserve (United States) and the Swiss National Bank to increase the liquidity of the market [44]. The breaking of time reversion symmetry in this case is consequence of several changes of price in one direction, similar to what happens during the Japanese government intervention; however, while in the Japanese government intervention case the price movements are responses to the changes of large magnitude by few traders and are against to the general tendency of the mid-quote, in this case of the announcement of the coordinated intervention the price movements are in accordance with the mid-quote tendency, suggesting trend-following behavior (see Fig 8-c). The picture suggested by this analysis is, therefore, that the sign dynamics of price change in the foreign exchange market is ordinarily time reversible, but there are rare occasions, either by direct external interference or reaction to important news, in which we detect time irreversible intervals.

## Final remarks

In this paper, we extended the concept of statistical symmetry by using transformations on probability distributions; we defined two different statistical symmetries, namely the the independence and reversion symmetries, and related them, respectively, to the mutual information and to the entropy production by using the Kullback-Leibler divergence as a measure of asymmetry. We then presented a hypothesis test based on Markov model to evaluate if a binary sign sequence presents a given statistical symmetry or not and applied it to real data of the foreign exchange market.

We analyzed the time series of the sign of price change of the USD/JPY mid-quote during nine weeks of October and November in 2011 and could describe different periods of the market according to their independence and time reversibility. We focused on the special event of the Japanese government intervention in the market and could characterize its two phases with different symmetries: The period of increase in the mid-quote as independence symmetric and the period of almost constant mid-quote as time reversion symmetric, but independence asymmetric. As a particular result, we found that the market in terms of direction of price change is essentially time reversible but this symmetry is broken in the presence of strong external influence, e.g., interventions.

We commented that the time reversion symmetry can be connected to the concept of entropy production: The entropy of a system changes (increases, for isolated system) when this symmetry is broken. Although we did not specify the system related to the market and thus did not define entropy for it, the results on the time reversion symmetry for the foreign exchange market data suggests that the breaking of this symmetry occurs when the market system is under an irreversible process, characterized by a quick change of the state of the market, seen in the abrupt change of the average mid-quote due to sequences of price movements in the same direction. Further investigations in this direction can provide a better understanding of the market in terms of entropy.

The analysis performed here used binary sign sequences and considered only pairs of symbols, being restricted to the simple binary sign Markov process. Nevertheless, keeping the information of the direction price change, we could perform a characterization of the foreign exchange market, in particular when under external influences. However, the concept of statistical symmetry is more general; it can be used for the theoretical analysis of stochastic process models and to characterize processes encoded in time series in order to extract useful information from big data of diverse fields. In future works, we intend to extend the framework in order to be able to work with more than 2 symbols, useful, for instance, in the study of DNA sequences (4 symbols); in the field of econophysics, we will be able to analyze price time series that also contains information about the magnitude of price changes and establish relationships between the statistical symmetries and existing market models.

## Supporting information

S1 Appendix Combinatorial expression for the number of binary sequences with given numbers of pairs. (PDF)

S2 Appendix Normal approximation for the probability distribution of the number of pairs in a stationary Markov process. (PDF)

S3 Appendix Statistical symmetries analysis on Markov process simulations. (PDF)

S4 Appendix Independence statistical symmetry for binary sequences and the Wald–Wolfowitz runs test. (PDF)

S5 Appendix Auto mutual information for sign time series from foreign exchange data and Markov process assumption. (PDF)
