# Supplementary material for: Detection of statistical asymmetries in non-stationary sign time series: Analysis of foreign exchange data
Source: PLoS One. 2017 May 18;12(5):e0177652. doi: 10.1371/journal.pone.0177652 (PMC5436817; doi:10.1371/journal.pone.0177652)
Supplement: S1 Appendix — (PDF) [file pone.0177652.s001.pdf]

Detection of statistical asymmetries in non-stationary sign time series: Analysis of foreign exchange data

Arthur Matsuo Yamashita Rios de Sousa, Hideki Takayasu, Misako Takayasu

Supporting information

S1 Appendix. Combinatorial expression for the number of binary sequences with given numbers of pairs.

Consider binary sequences of length w composed by symbols $(+)$ and $(-)$. We would like an expression that inform us the number γ of such sequences that contain a given quantity of pairs $(++)$, $(+-)$, $(-+)$ and $(--)$.

The number of different pairs in a given sequence are not independent. To check this fact, let us first take closed sequences. In closed sequences, for each symbol $(+)$ we have a pair $(++)$ or a pair $(-+)$ formed with the symbol on its left and a pair $(+,+)$ or a pair $(+-)$ on its right. Summing all the occurrences, we obtain the relationship between the number N_+ of symbols $(+)$ and the number N^C of each pair in closed sequences:

$$N_+ = N^C_{++} + N^C_{+-}, \tag{1}$$

$$N_+ = N^C_{++} + N^C_{-+}. \tag{2}$$

Similarly, for each symbol $(-)$ we have the corresponding pairs, leading to:

$$N_- = N^C_{--} + N^C_{-+}, \tag{3}$$

$$N_- = N^C_{--} + N^C_{+-}. \tag{4}$$

We also have the restriction of the sequence length w:

$$N_+ + N_- = w. \tag{5}$$

Let us define domain $(+)$ in a closed sequence as a maximal subsequence composed only by symbols $(+)$. For each domain $(+)$ we have a pair $(+-)$ and a pair $(-+)$ in each of its extremities. Then, the number g^C_+ of domains $(+)$ in closed sequences:

$$g^C_+ = N^C_{+-} = N^C_{-+}, \tag{6}$$

which can also be expressed as:

$$g^C_+ = N_+ - N^C_{++}. \tag{7}$$

In the same way, for domains $(-)$ in closed sequences:

$$g_-^C = N_- - N_{--}^C. \tag{8}$$

In order to study open sequences, we break the closed one in some point, reducing the number of the corresponding pair by 1:

$$N_{++} = N_{++}^C - \varepsilon(+,+), \tag{9}$$

$$N_{+-} = N_{+-}^C - \varepsilon(+,-), \tag{10}$$

$$N_{-+} = N_{-+}^C - \varepsilon(-,+), \tag{11}$$

$$N_{--} = N_{--}^C - \varepsilon(-,-), \tag{12}$$

where $\varepsilon(i,j)$ indicates the boundary of the sequence (the first symbol s_1 and the last symbol s_w), with $i,j = (+)$ or $(-)$:

$$\varepsilon(i,j) = \begin{cases} 1; & \text{if } s_1 = j \text{ and } s_w = i \\ 0; & \text{otherwise} \end{cases}. \tag{13}$$

Note that in the notation $\varepsilon(i,j)$, i represents the last symbol and j, the first one.

Substituting in the expression for closed sequences, we obtain the relations for open sequences:

$$N_+ = N_{++} + N_{+-} + \varepsilon(+,+) + \varepsilon(+,-), \tag{14}$$

$$N_+ = N_{++} + N_{-+} + \varepsilon(+,+) + \varepsilon(-,+), \tag{15}$$

$$N_- = N_{--} + N_{-+} + \varepsilon(-,-) + \varepsilon(-,+), \tag{16}$$

$$N_- = N_{--} + N_{+-} + \varepsilon(-,-) + \varepsilon(+,-). \tag{17}$$

The numbers of domains g_+ and g_- remain the same, except when $\varepsilon(+,+) = 1$ or $\varepsilon(-,-) = 1$:

$$g_+ = g_+^C + \varepsilon(+,+) = N_+ - N_{++}, \tag{18}$$

$$g_- = g_-^C + \varepsilon(-,-) = N_- - N_{--}. \tag{19}$$

Fig 1 depicts an example of open binary sign sequence and the involved quantities.

(+ + - + - - + + + - - + + - + + + + - - - - + + - -)

Figure 1: **Example of open binary sign sequence.** Open binary sign sequence of length $w = 26$ with $N_+ = 14$ symbols $(+)$, $N_- = 12$ symbols $(-)$, the first symbol $s_1 = (+)$, the last symbol $s_w = (-)$ and $\varepsilon(-,+) = 1$. The numbers of each pair are: $N_{++} = 8$, $N_{+-} = 6$, $N_{-+} = 5$ and $N_{--} = 6$. The number of domains: $g_+ = 6$ and $g_- = 6$.

Having the number of domains in open sequences expressed in terms of the number of pairs, we can compute the number γ, since it is equivalent to the number of binary sequences with g_+ domains $(+)$ and g_- domains $(-)$ (for each sequence boundary). Using the multiplicative principle, this number is equal to: "The number of ways to group N_+ indistinguishable objects in g_+ non-empty distinguishable groups" multiplied by "the number of ways to group N_- indistinguishable objects in g_- non-empty distinguishable groups".

Call ξ the number of ways to group n indistinguishable objects in k non-empty distinguishable groups. Let us use the balls and bars graphical scheme in Fig 2 to obtain it.

We have two kinds of elements: Balls attached to a bar and single balls. Since we want k groups, we have $k-1$ elements of the first type and $n-1-(k-1) = n-k$ elements of the second type. ξ is the number of permutations of those elements:

$$\xi = \frac{[(n-k)+(k-1)]!}{(n-k)!(k-1)!} = \binom{n-1}{k-1}. \tag{20}$$

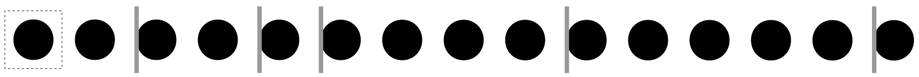

Figure 2: **Balls and bars graphical scheme to count the number of ways to group n indistinguishable objects in k non-empty distinguishable groups.** Balls represent the objects and bars are the separators of groups. The problem is equivalent to the permutations of two types of elements: Free objects and pairs object-separator, with the first object fixed because of the non-empty groups restriction.

Therefore:

$$\gamma = \binom{N_+ - 1}{g_+ - 1}\binom{N_- - 1}{g_- - 1} = \binom{N_+ - 1}{N_{++}}\binom{N_- - 1}{N_{--}}, \tag{21}$$

for one of the four types of boundary.

If we represent γ using the numbers N_+ and N_{++}:

$$\gamma = \binom{N_+ - 1}{N_{++}}\binom{w - N_+ - 1}{N_+ - N_{++} - \varepsilon(+,+) + \varepsilon(-,-) - 1}. \tag{22}$$

Note that the derived expression for γ is valid only for sequences with at least one of each symbol. For the trivial cases, with $N_+ = 0$ or $N_+ = w$, we only have one sequence for each: $\gamma = 1$.
