# Supplementary material for: Detection of statistical asymmetries in non-stationary sign time series: Analysis of foreign exchange data
Source: PLoS One. 2017 May 18;12(5):e0177652. doi: 10.1371/journal.pone.0177652 (PMC5436817; doi:10.1371/journal.pone.0177652)
Supplement: S2 Appendix — (PDF) [file pone.0177652.s002.pdf]

Detection of statistical asymmetries in non-stationary sign time series: Analysis of foreign exchange data

Arthur Matsuo Yamashita Rios de Sousa, Hideki Takayasu, Misako Takayasu

Supporting information

S2 Appendix. Normal approximation for the probability distribution of the number of pairs in a stationary Markov process.

The probability $\mathcal{P}$ of a stationary sign binary Markov process $\{\mu, \nu\}$ to generate a sequence with numbers of pairs N_{++}, N_{+-}, N_{-+}, N_{--}, the first symbol s_1 and the last symbol s_w, given length w and parametrized using N_+ and N_{++}, is given by:

$$\mathcal{P} = \binom{N_+ - 1}{N_{++}}\binom{N_- - 1}{N_{--}} \times \frac{1}{\mu+\nu}(1-\mu)^{N_{++}}\mu^{N_+ - N_{++} - \varepsilon(+,+) + \varepsilon(-,-)}\nu^{N_- - N_{--} + \varepsilon(+,+) - \varepsilon(-,-)}(1-\nu)^{N_{--}}, \quad (1)$$

for $0 < N_+ < w$, with $N_- = w - N_+$ and $N_{--} = w - 2N_+ + N_{++} + \varepsilon(+,+) - \varepsilon(-,-)$.

The above expression can be rearranged in order to explicit two terms resembling binomial distributions:

$$\mathcal{P} = \frac{\nu^{1+\varepsilon(+,+)-\varepsilon(-,-)}\mu^{1-\varepsilon(+,+)+\varepsilon(-,-)}}{\mu+\nu} \times \binom{N_+ - 1}{N_{++}}(1-\mu)^{N_{++}}\mu^{N_+ - N_{++}}\binom{N_- - 1}{N_{--}}(1-\nu)^{N_{--}}\nu^{N_- - N_{--}}. \quad (2)$$

Now we use the normal approximation for the binomial distribution:

$$\binom{n}{k}p^k(1-p)^{n-k} \approx \frac{1}{\sqrt{2\pi np(1-p)}} exp\left(-\frac{(k-np)^2}{2np(1-p)}\right). \quad (3)$$

And finally we obtain the approximate expression for the $\mathcal{P}$:

$$\mathcal{P} \approx \frac{\nu^{1+\varepsilon(+,+)-\varepsilon(-,-)}\mu^{1-\varepsilon(+,+)+\varepsilon(-,-)}}{\mu+\nu} \times \frac{1}{\sqrt{2\pi(N_+ - 1)\mu(1-\mu)}} exp\left(-\frac{[N_{++} - (N_+ - 1)(1-\mu)]^2}{2(N_+ - 1)\mu(1-\mu)}\right) \times \frac{1}{\sqrt{2\pi(N_- - 1)\nu(1-\nu)}} exp\left(-\frac{[(N_{--} - (N_- - 1)(1-\nu)]^2}{2(N_- - 1)\nu(1-\nu)}\right). \quad (4)$$
