# Supplementary material for: Detection of statistical asymmetries in non-stationary sign time series: Analysis of foreign exchange data
Source: PLoS One. 2017 May 18;12(5):e0177652. doi: 10.1371/journal.pone.0177652 (PMC5436817; doi:10.1371/journal.pone.0177652)
Supplement: S3 Appendix — (PDF) [file pone.0177652.s003.pdf]

Detection of statistical asymmetries in non-stationary sign time series: Analysis of foreign exchange data

Arthur Matsuo Yamashita Rios de Sousa, Hideki Takayasu, Misako Takayasu

Supporting information

S3 Appendix. Statistical symmetries analysis on Markov process simulations.

The statistical symmetries analysis presented in this paper consists in a hypothesis test based on a stationary binary Markov process. Aiming to evaluate the performance of the test and its dependence on the sequence length w, we simulate and analyze stationary binary Markov chains of different lengths w with known properties.

First, we estimate the cumulative distribution functions of $\mathcal{P}$ (Eq 19 in the section *Analysis of sign time series and hypothesis test*) when we test independence and odd reversion symmetries for stationary binary Markov process with different parameters μ and ν but fixed sequence length $w = 1000$, as described in the section *Analysis of sign time series and hypothesis test.* We call independence symmetric a process generated with parameters such that $\mu + \nu = 1$, odd reversion symmetric a process generated with parameters such that $\mu = \nu$, and independence and odd reversion asymmetric a process generated with parameters obeying neither the previous conditions. Fig 1 shows the cumulative distribution functions for: independence symmetry test (Fig 1-a-1) and odd reversion symmetry test (Fig 1-a-2) on independence symmetric processes ($\mu + \nu = 1$); independence symmetry test (Fig 1-b-1) and odd reversion symmetry test (Fig 1-b-2) on odd reversion symmetric processes ($\mu = \nu$); and independence symmetry test (Fig 1-c-1) and odd reversion symmetry test (Fig 1-c-2) on independence and odd reversion asymmetric processes ($\nu = 0.5$). We obtain different functional forms depending on whether we test a given symmetry on a corresponding symmetric process (Fig 1-a-1 and 1-b-2) or on an asymmetric process (Fig 1-a-2, 1-b-1, 1-c-1 and 1-c-2), having for the latter a high cumulative probability for low values of $\mathcal{P}$. Focusing on the particular value $\mathcal{P} = 10^{-7}$, for symmetry test on symmetric process, the probability of $\mathcal{P} < 10^{-7}$ is below 10^{-4} and for symmetry test on asymmetric process, this probability is above 10^{-4}, becoming higher for more asymmetric processes (μ and/or ν distancing 0.5) - it approaches 1 for μ and/or ν around 0.4. For the statistical symmetries analysis, taking $z = 10^{-7}$ as threshold, it means that the probability of evaluating a symmetric interval as asymmetric (type I error) is below 10^{-4} and the probability of evaluating an asymmetric interval as symmetric (type II error) is below $1 - 10^{-4}$, a large value if the process presents weak asymmetry, i.e., $\mu + \nu \approx 1$ for independent symmetric process or $\mu \approx \nu$ for odd reversion symmetric process, but being around 0.8 if the process presents mild asymmetry, with μ and/or ν around 0.45, and approaching 0 for processes with strong asymmetry.

We also examine the dependence of the cumulative distribution functions of $\mathcal{P}$ on the sequence length w by performing a similar analysis as previous, but varying w from 200 to 2000. Fig 2 shows the results: independence symmetry test (Fig 2-a-1) and odd reversion symmetry test (Fig 2-a-2) on the uniform process ($\mu = \nu = 0.5$); independence symmetry test (Fig 2-b-1) and odd reversion symmetry test (Fig 2-b-2) on an independence symmetric process with $\mu = 0.3$ and $\nu = 0.7$; and independence symmetry test (Fig 2-c-1) and odd reversion symmetry test (Fig 2-c-2) on a odd reversion symmetric process with $\mu = 0.3$ and $\nu = 0.3$. Here, we also have two types of structures: Observing the value $\mathcal{P} = 10^{-7}$, for symmetry test on symmetric process, the probability of $\mathcal{P} < 10^{-7}$ increases with w but it is approximately below 10^{-4} for all considered lengths (Fig 2-a-1, 2-a-2, 2-b-1 and 2-c-2) and for symmetry test on asymmetric process, this probability is above 10^{-4}, becoming quickly higher for larger w (Fig 2-b-2 and 2-c-1). For the statistical symmetries analysis considering window sizes, the observation of asymmetry of an interval in all window sizes increases our belief that this interval is indeed asymmetric.

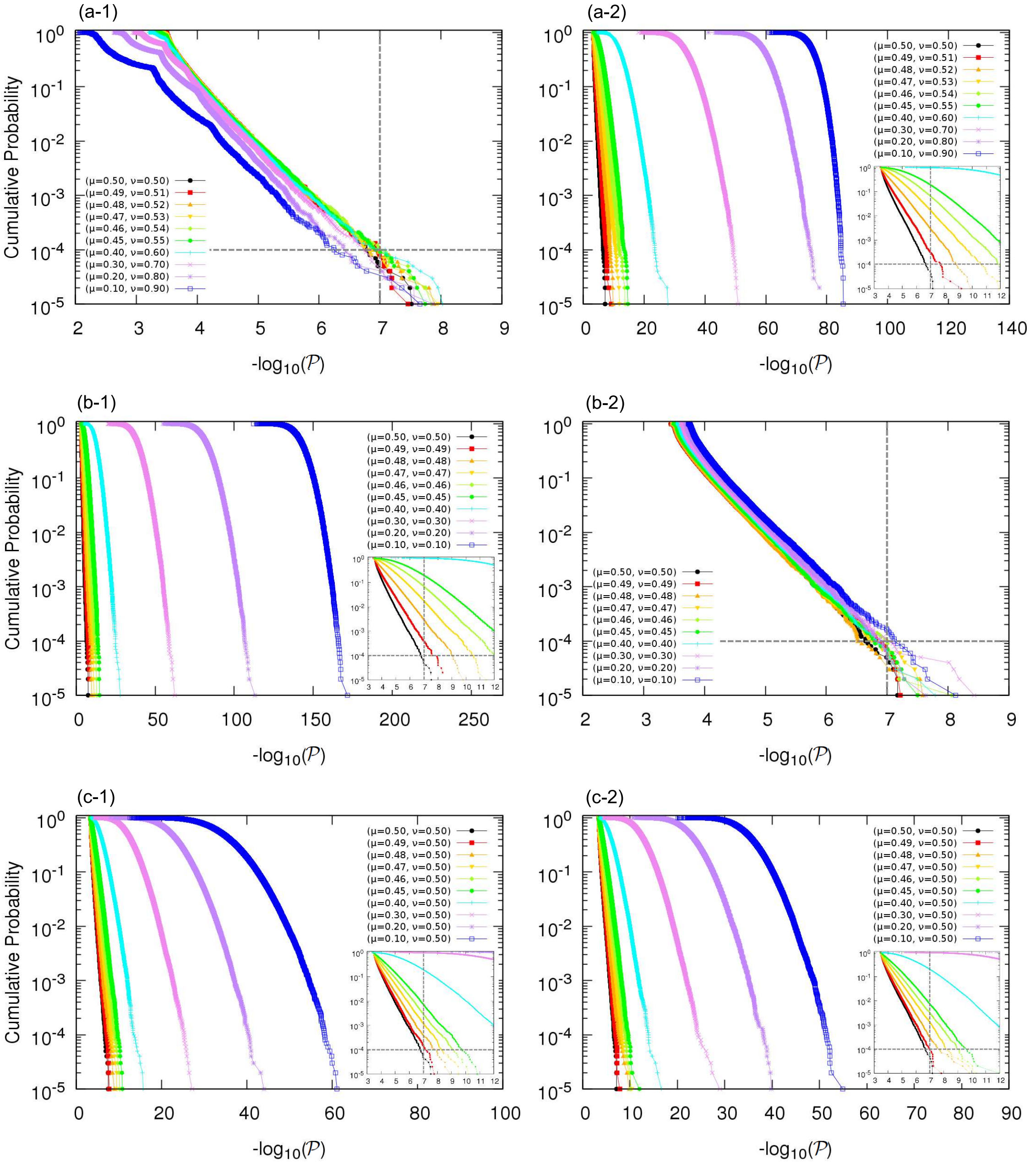

Figure 1: **Estimated cumulative distribution functions of $\mathcal{P}$ when independence and odd reversion symmetries are tested for simulated stationary binary Markov process with fixed sequence length $w = 1000$ but different parameters μ and ν.** Independence symmetry test (a-1) and odd reversion symmetry test (a-2) on independence symmetric processes ($\mu + \nu = 1$); independence symmetry test (b-1) and odd reversion symmetry test (b-2) on odd reversion symmetric processes ($\mu = \nu$); and independence symmetry test (c-1) and odd reversion symmetry test (c-2) on independence and odd reversion asymmetric processes ($\nu = 0.5$). Note that the process $\{\mu = 0.5, \nu = 0.5\}$ is both independence and odd reversion symmetric. Inset figures detail the cumulative distribution functions for large $\mathcal{P}$.

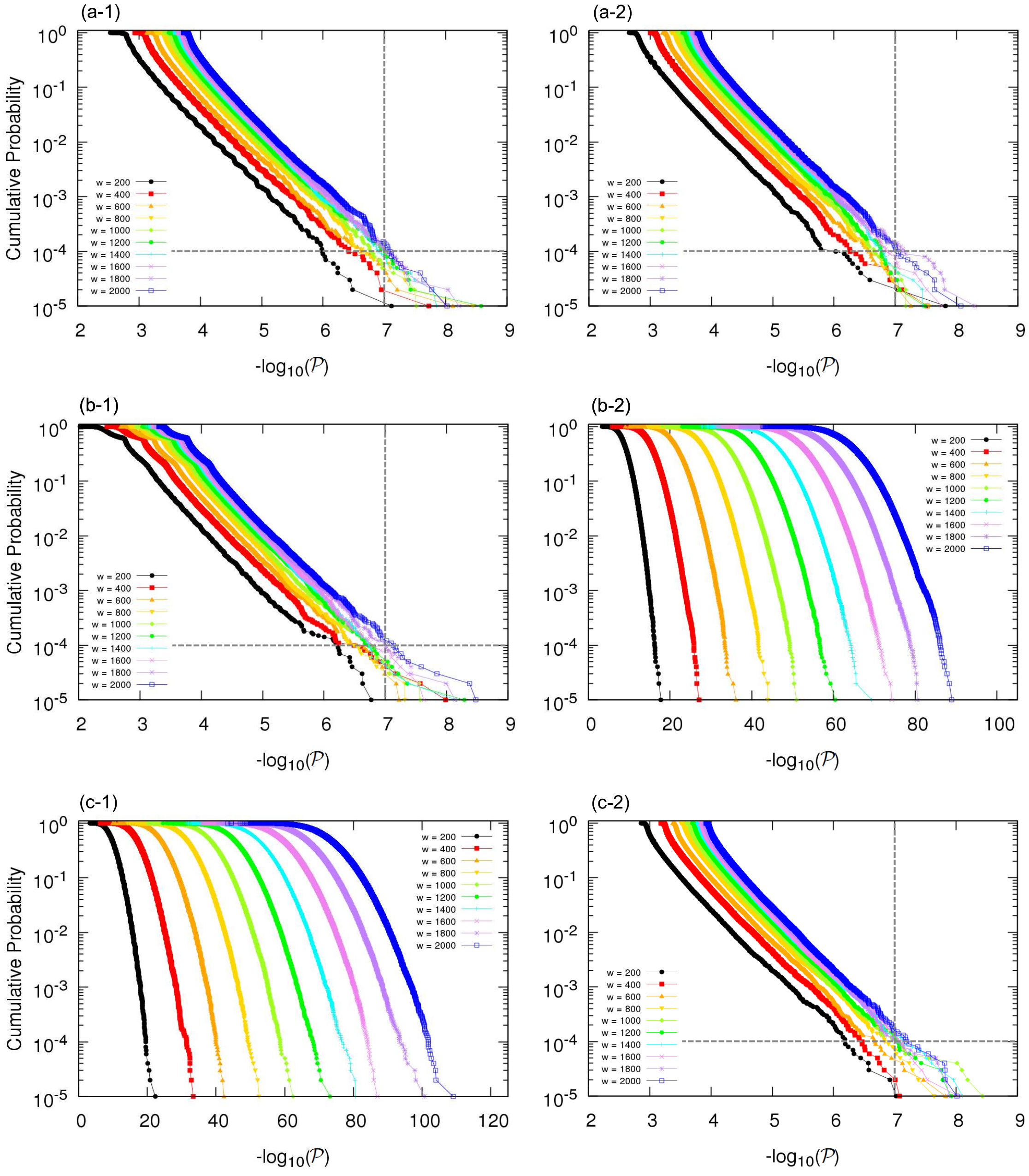

Figure 2: **Estimated cumulative distribution functions of $\mathcal{P}$ when independence and odd reversion symmetries are tested for simulated stationary binary Markov process varying the sequence length w.** Independence symmetry test (a-1) and odd reversion symmetry test (a-2) on the uniform process ($\mu = \nu = 0.5$); independence symmetry test (b-1) and odd reversion symmetry test (b-2) on an independence symmetric process with $\mu = 0.3$ and $\nu = 0.7$; and independence symmetry test (c-1) and odd reversion symmetry test (c-2) on a odd reversion symmetric process with $\mu = 0.3$ and $\nu = 0.3$.
