# Supplementary material for: Detection of statistical asymmetries in non-stationary sign time series: Analysis of foreign exchange data
Source: PLoS One. 2017 May 18;12(5):e0177652. doi: 10.1371/journal.pone.0177652 (PMC5436817; doi:10.1371/journal.pone.0177652)
Supplement: S4 Appendix — (PDF) [file pone.0177652.s004.pdf]

Detection of statistical asymmetries in non-stationary sign time series: Analysis of foreign exchange data

Arthur Matsuo Yamashita Rios de Sousa, Hideki Takayasu, Misako Takayasu

Supporting information

S4 Appendix. Independence statistical symmetry for binary sequences and the Wald–Wolfowitz runs test.

The Wald–Wolfowitz runs test is a non-parametric statistical test to verify a randomness hypothesis in binary sequences. It is a hypothesis test with null hypothesis that the elements of the sequence are mutually independent and identically distributed. It uses the distribution of runs, being a run a maximal subsequence composed by elements of the same type; the domains (+) and (−) defined in supporting information *S4 Appendix* correspond to runs and, denoting the number of runs by r, we have:

$$r = g_+ + g_-. \tag{1}$$

The distribution of runs for independence hypothesis is:

$$P(r|N_+, N_-) = \begin{cases} \frac{2\binom{N_+-1}{\frac{r}{2}-1}\binom{N_--1}{\frac{r}{2}-1}}{\binom{N_++N_-}{N_+}}; & r \text{ even} \\ \\ \frac{\binom{N_+-1}{\frac{r-1}{2}}\binom{N_--1}{\frac{r-1}{2}-1}+\binom{N_+-1}{\frac{r-1}{2}-1}\binom{N_--1}{\frac{r-1}{2}}}{\binom{N_++N_-}{N_+}}; & r \text{ odd} \end{cases}, \tag{2}$$

which can be approximated to a normal distribution for practical purposes.

We show here how the runs test is related to the hypothesis test for independence statistical symmetry in this paper. Let us work with the boundary indicators $\varepsilon(s_w, s_1)$ and separate r in g_+ and g_-:

$$P(r, s_1, s_w|N_+, N_-) = \frac{\binom{N_+-1}{g_+-1}\binom{N_--1}{g_--1}}{\binom{N_++N_-}{N_+}}, \tag{3}$$

with $g_+ + \varepsilon(+,+) = g_- + \varepsilon(-,-)$.

Let us select the Markov model. Since we can express the number of pairs in a binary sequence in terms of number of domains, the probability of a stationary binary Markov process $\{\mu, \nu\}$ to generate a sequence with N_{++}, N_{+-}, N_{-+}, N_{--}, s_1, s_w given length w is equivalent to the probability of the process to generate N_+, N_-, g_+, g_-, s_1, s_w given length w (for $0 < N_+ < w$):

$$P(N_+, g_+, s_1, s_w|\{\mu, \nu\}; w) = \binom{N_+-1}{g_+-1}\binom{N_--1}{g_--1}\frac{(1-\mu)^{N_+-g_+}(\mu)^{g_+}(\nu)^{g_-}(1-\nu)^{N_--g_-}}{\mu+\nu}, \tag{4}$$

with $N_- = w - N_+$ and $g_- = g_+ + \varepsilon(+,+) - \varepsilon(-,-)$.

The conditional probability for given $N(+)$ (and also $N(-)$ because the length w is fixed) is obtained as:

$$P(g_+, s_1, s_w|\{\mu, \nu\}; N_+, N_-) = \frac{P(N_+, g_+, s_1, s_w|\{\mu, \nu\}; w)}{P(N_+|\{\mu, \nu\}; w)}, \tag{5}$$

where

$$P(N_+|\{\mu, \nu\}; w) = \sum_{s_1, s_w} \sum_{g_+} P(N_+, g_+, s_1, s_w|\{\mu, \nu\}; w). \tag{6}$$

This conditional probability distribution is equal to the one used for the runs test if we take independent Markov process, $\mu + \nu = 1$, in which all sequences with same N_+ and N_- have the same probability.

In this case, we have for independent binary Markov process:

$$P(N_+, g_+, s_1, s_w|\{\mu + \nu = 1\}; w) = \binom{N_+ - 1}{g_+ - 1}\binom{N_- - 1}{g_- - 1}\nu^{N_+}\mu^{N_-}, \tag{7}$$

and

$$P(N_+|\{\mu + \nu = 1\}; w) = \binom{N_+ + N_-}{N_+}\nu^{N_+}\mu^{N_-}. \tag{8}$$

We, therefore, recover the runs test distribution by taking the conditional probability distribution give the number of each element and considering the independent case:

$$P(g_+, s_1, s_w|\{\mu + \nu = 1\}; N_+, N_-) = \frac{\binom{N_+ - 1}{g_+ - 1}\binom{N_- - 1}{g_- - 1}}{\binom{N_+ + N_-}{N_+}}. \tag{9}$$

Since both probability distributions are directly related, it is expected that the result of the Wald-–Wolfowitz runs test will be in general equivalent to the proposed hypothesis test for the independence symmetry.
