# Supplementary material for: Detection of statistical asymmetries in non-stationary sign time series: Analysis of foreign exchange data
Source: PLoS One. 2017 May 18;12(5):e0177652. doi: 10.1371/journal.pone.0177652 (PMC5436817; doi:10.1371/journal.pone.0177652)
Supplement: S5 Appendix — (PDF) [file pone.0177652.s005.pdf]

Detection of statistical asymmetries in non-stationary sign time series: Analysis of foreign exchange data

Arthur Matsuo Yamashita Rios de Sousa, Hideki Takayasu, Misako Takayasu

Supporting information

S5 Appendix. Auto mutual information for sign time series from foreign exchange data and Markov process assumption.

The mutual information measures the dependence between two random variables by directly examining their probability distributions. The mutual information $I(X;Y)$ between two random variables X and Y is given by:

$$I(X;Y) = \sum_{x}\sum_{y} p(x,y) log \frac{p(x,y)}{p(x)p(y)}, \tag{1}$$

where $p(x,y)$ is the joint probability distribution of X and Y and $p(x)$, $p(y)$ are the marginal distributions.

In order to verify the validity of assumption that the signs of the price movement can be described by a Markov process, we use the mutual information to access the dependence of the binary sign time series from foreign exchange data on its own past steps, by comparing the series with itself for different time shifts.

Fig 1 shows the estimated auto mutual information of the sign time series from the data described in this paper (USD/JPY from 2011, October, 30th to 2011, November, 5th) for different time shifts and sampling times Δt. For the original sampling time $\Delta t = 0.1s$, the mutual information presents an oscillatory behavior. But, from sampling time $\Delta t = 0.4s$, the mutual information goes to zero after few time shifts, justifying the approximation to a Markov model at $\Delta t = 0.4s$.

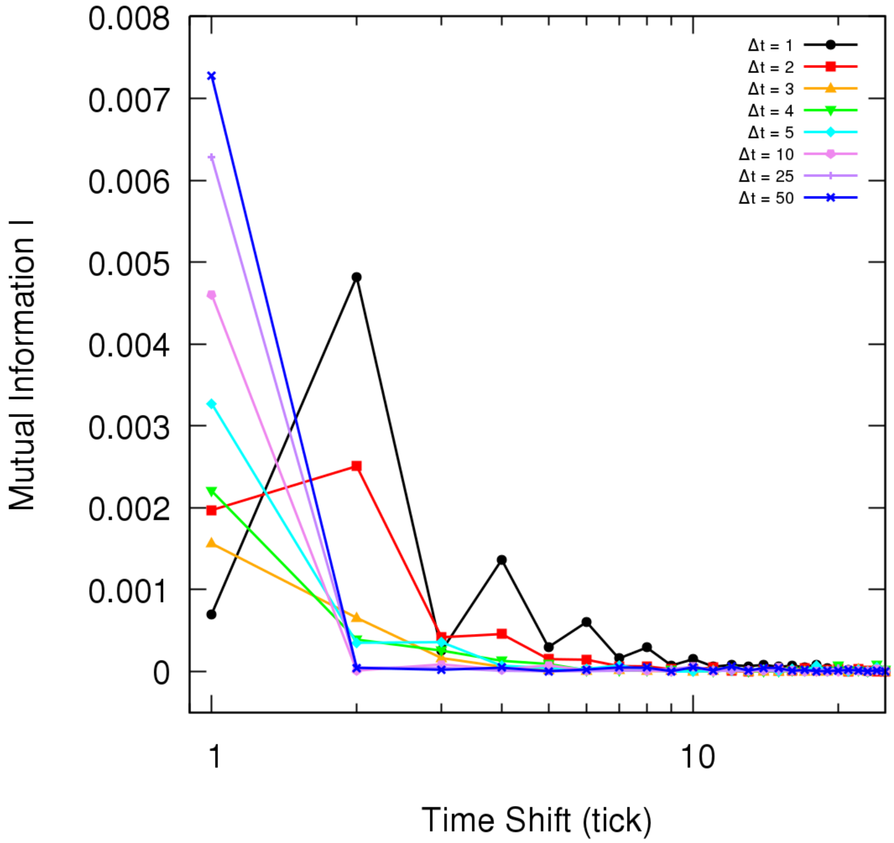

Figure 1: **Estimated auto mutual information of the sign time series from the USD/JPY foreign exchange data (week from 2011, October, 30th to 2011, November, 5th) for different time shifts and sampling times Δt.** From sampling time $\Delta t = 0.4s$, the mutual information goes to zero after few time shifts.
